# *Staphylococcus aureus* Infections in Malaysia: A Review of Antimicrobial Resistance and Characteristics of the Clinical Isolates, 1990–2017

**DOI:** 10.3390/antibiotics8030128

**Published:** 2019-08-26

**Authors:** Ainal Mardziah Che Hamzah, Chew Chieng Yeo, Suat Moi Puah, Kek Heng Chua, Ching Hoong Chew

**Affiliations:** 1 Faculty of Health Sciences, Universiti Sultan Zainal Abidin, Kuala Nerus 21300, Terengganu, Malaysia; 2 Faculty of Medicine, Universiti Sultan Zainal Abidin, Kuala Terengganu 20400, Terengganu, Malaysia; 3 Department of Biomedical Science, Faculty of Medicine, University of Malaya, Kuala Lumpur 50603, Malaysia

**Keywords:** Antimicrobial resistance, community-associated (CA), hospital-associated (HA), *Staphylococcus aureus*, macrolide-lincosamide-streptogramin B (MLS_B_), Malaysian clinical isolates, methicillin-resistance *S. aureus* (MRSA), methicillin-susceptible *S. aureus* (MSSA), staphylococcal cassette chromosome *mec* (SCC*mec*) type, sequence types (STs)

## Abstract

*Staphylococcus aureus* is an important nosocomial pathogen and its multidrug resistant strains, particularly methicillin-resistant *S. aureus* (MRSA), poses a serious threat to public health due to its limited therapeutic options. The increasing MRSA resistance towards vancomycin, which is the current drug of last resort, gives a great challenge to the treatment and management of MRSA infections. While vancomycin resistance among Malaysian MRSA isolates has yet to be documented, a case of vancomycin resistant *S. aureus* has been reported in our neighboring country, Indonesia. In this review, we present the antimicrobial resistance profiles of *S. aureus* clinical isolates in Malaysia with data obtained from the Malaysian National Surveillance on Antimicrobial Resistance (NSAR) reports as well as various peer-reviewed published records spanning a period of nearly three decades (1990–2017). We also review the clonal types and characteristics of Malaysian *S. aureus* isolates, where hospital-associated (HA) MRSA isolates tend to carry staphylococcal cassette chromosome *mec* (SCC*mec*) type III and were of sequence type (ST)239, whereas community-associated (CA) isolates are mostly SCC*mec* type IV/V and ST30. More comprehensive surveillance data that include molecular epidemiological data would enable further in-depth understanding of Malaysian *S. aureus* isolates.

## 1. Introduction

*Staphylococcus aureus* is a frequently encountered Gram-positive nosocomial pathogen that is associated with a wide array of diseases, ranging from simple skin infection to more serious and potentially life-threatening infections such as infective endocarditis and toxic shock syndrome [1,2]. Its ability to rapidly develop and acquire antimicrobial resistance has led to the emergence of multidrug resistant strains such as methicillin-resistant *S. aureus* (MRSA) [1]. The MRSA strains have become endemic in most hospitals worldwide, including in Asia [3], and are a treatment challenge to physicians.

In their 2014 global report, the World Health Organization (WHO) listed MRSA as one of the seven pathogens of international concern and that has been associated with a high number of mortality and septic shock cases compared to MSSA [1]. In Malaysia, the national prevalence rate of MRSA among *S. aureus* clinical isolates ranged from 17.2% to 28.1%, with the rates recorded at 18% and 19.8% for the years 2016 and 2017, respectively [4,5]. At present, vancomycin remains the drug of last resort for the treatment of MRSA infections [6]. However, reports regarding MRSA strains that had developed resistance to vancomycin have emerged in many parts of the world, with the first of such strains reported in the United States almost two decades ago [7]. Nevertheless, the occurrence of such strains, known as vancomycin-resistant *S. aureus* (VRSA), is not as abundant as MRSA. To date, in the United States alone, 14 VRSA strains have been reported, of which only one is community-associated (CA) while the rest are hospital-associated (HA) strains [8]. While VRSA is still considered as a rare bacterium that has yet to achieve endemic status like MRSA, the possibility of VRSA isolates becoming widely disseminated must not be ignored, as it could cause serious implications for public health. There has been no published report of vancomycin resistance among Malaysian MRSA isolates so far. However, a VRSA strain with a vancomycin MIC value of ≥ 32 µg/mL has been isolated from a patient in the neighboring country, Indonesia [9]. Thus, we believe VRSA strains could also be detected in Malaysia sooner or later.

The purpose of this review is to provide a general overview of the antimicrobial resistance trends of clinical *S. aureus* isolates in Malaysia obtained from various published reports of individual studies as well as the Malaysian National Surveillance on Antimicrobial Resistance (NSAR) annual reports. The molecular mechanism of antibiotic resistance and other characteristics of the *S. aureus* isolates, such as their sequence types (STs) and other molecular epidemiological markers, are also presented. 

## 2. Antibiotic Susceptibility Profiles

Malaysian NSAR is an active antimicrobial surveillance program which is published online annually and encompasses various bacterial pathogens including *S. aureus*. NSAR is under the Malaysian One Health Antimicrobial Resistance (MyOHAR) program, which is hosted by the Institute for Medical Research (IMR) starting from year 2003 (with the exception of year 2006) and involves clinical isolates tested by hospital microbiology laboratories throughout Malaysia (https://www.imr.gov.my/MyOHAR/index.php/site/archive_rpt). The antimicrobials tested for *S. aureus* in the NSAR reports vary from year to year but usually include erythromycin, gentamicin, co-trimoxazole, rifampicin, fusidic acid, and clindamycin, besides many others. Most Malaysian studies, as well as NSAR data, tend to focus more on MRSA isolates rather than methicillin-susceptible *S. aureus* (MSSA) isolates, thus data concerning antimicrobial resistance among Malaysian MSSA isolates are rather limited. In the guidelines published by the Clinical and Laboratories Standard Institute (CLSI), a total of 43 antibiotics from 16 antimicrobial classes were listed for *S. aureus* (Table 1) [10]. The European Centre for Disease Prevention and Control (ECDC) in conjunction with the Centers for Disease Control and Prevention (CDC) of the United States also published a list of antimicrobials recommended for susceptibility testing of *S. aureus* isolates, which included 22 antibiotics from 17 antimicrobial classes, including three agents which were not listed in the CLSI guideline, namely fusidic acid, tigecycline, and fosfomycin [11]. Nevertheless, the guideline published by the European Committee on Antimicrobial Susceptibility Testing (EUCAST) covered as many as 16 antimicrobial classes encompassing 38 antimicrobial agents, and includes the three agents that are not covered by CLSI [12]. 

Thus far, a total of 11 individual studies and 12 NSAR reports regarding the antimicrobial resistance profiles of clinical *S. aureus* in Malaysia were documented from 1990 to 2017. The details regarding the framework of data from these NSAR reports as well as individual studies are shown in Table 2.

### 2.1. β-Lactams (Penicillin, Oxacilin, Cefoxitin, and Cefoperazone)

Resistance towards β-lactam antimicrobials among MRSA isolates in Malaysia has always been high due to the nature of MRSA itself, where the presence of the *mecA* gene that encodes the penicillin binding protein 2a (PBP 2a) confers resistance to β-lactams through a mechanism in which the drug binding affinity is affected [34]. An early report involving 539 MRSA isolates from Hospital Kuala Lumpur (HKL) between 1990 and 1991 recorded full resistance to penicillin [13]. Thirty two MRSA isolates obtained between 2006 and 2007 from an unnamed tertiary hospital were also fully resistant to penicillin, oxacillin, and cefoxitin [17]. Similarly, complete resistance was also reported for oxacillin and penicillin in 66 MRSA isolates that were collected from an unspecified teaching hospital in 2003, 2004, and 2007 [16] and 1979 MRSA isolates from Hospital Universiti Sains Malaysia (HUSM), obtained from 2002 until 2007 [15]. Similar observations were also recorded among 175 MRSA isolates collected between 2011 and 2012 from Hospital Raja Permaisuri Bainun (HRPB), KPJ Ipoh Specialist Hospital (KPJ), and Gribbles Pathology Ipoh (GP) (located at Ipoh, the capital city of Perak state on the west coast of peninsular Malaysia [21]), as well as 67 MRSA strains obtained from adult patients (above 16 years old) with bacteremia in University of Malaya Medical Centre (UMMC) in 2013 [22]. Ninety MRSA isolates were obtained between 2016 and 2017 from Hospital Sultanah Nur Zahirah (HSNZ), located at Kuala Terengganu, the state capital of Terengganu on the east coast of peninsular Malaysia also showed full resistance to penicillin, oxacillin and cefoxitin [23]. 

Cefoperazone is rarely used in Malaysia and thus there is a scarcity of data reporting the rates of cefoperazone resistance among Malaysian MRSA isolates. To the best of our knowledge, our recent study on isolates from HSNZ is the only report of cefoperazone resistance among Malaysian MRSA isolates, with the prevalence of resistance recorded at 86.7% for the 2016–2017 isolates [23]. 

Reports describing β-lactam resistance among Malaysian MSSA isolates are even scarcer. An early study reported an approximately 90% prevalence of resistance to penicillin and full susceptibility to oxacillin among 124 MSSA isolates from HUSM between March to August 2008 [19], while our recent study reported 84.4% and 5.5% prevalence of resistance to penicillin and oxacillin among 109 HSNZ MSSA isolates obtained in 2016 and 2017 [23].

### 2.2. Aminoglycosides (Gentamicin, Amikacin, Netilmicin, and Kanamycin)

**Figure 1:**
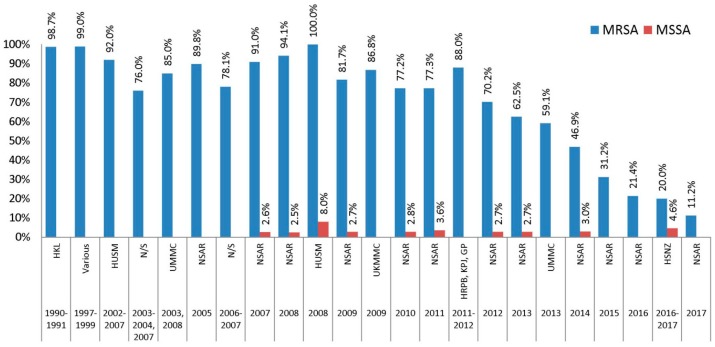
The prevalence of gentamicin resistance among Malaysian *S. aureus* isolates, 1990–2017. Data from the National Surveillance of Antibiotic Resistance (NSAR) reports, 2003–2005 [24], 2007 [26], 2008 [25], 2009 [27], 2010 [28], 2011 [29], 2012 [30], 2013 [31], 2014 [32], 2015 [33], 2016 [4], and 2017 [5]; Hospital Kuala Lumpur (HKL) between 1990 and 1991 [13]; Various, collected from ten hospitals throughout Malaysia between 1997 and 1999 [14]; Hospital Universiti Sains Malaysia (HUSM) between 2002 and 2007 [15] and in 2008 [19]; not specified (N/S) between 2003 and 2004 and in 2007 [16], and between 2006 and 2007 [17]; University of Malaya Medical Centre (UMMC) in 2003 and 2008 [18], and in 2013 [22]; Universiti Kebangsaan Malaysia Medical Centre (UKMMC) in 2009 [20]; Hospital Raja Permaisuri Bainun (HRPB), KPJ Ipoh Specialist Hospital (KPJ) and Gribbles Pathology Ipoh (GP) between 2011 and 2012 [21]; and Hospital Sultanah Nur Zahirah (HSNZ) between 2016 and 2017 [23].

Gentamicin resistance among Malaysian MRSA isolates was generally high in the earlier years of the NSAR reports but showed a declining trend towards recent years (Figure 1). In general, MSSA isolates tend to be much more susceptible to gentamicin as compared to MRSA isolates. Among the antibiotics in the aminoglycoside group, only gentamicin was frequently reported by NSAR, while amikacin resistance was only reported in 2007 and 2008, whereas netilmicin and kanamycin resistance were never reported. In the proposed CDC-ECDC criteria for *S. aureus*, only gentamicin is recommended to be tested for the aminoglycoside group [11].

Isolates obtained in the 1990s showed very high prevalence of gentamicin resistance (>98%) (Figure 1). The MRSA isolates collected between 2003 and 2009 in various studies as well as the NSAR reports showed a fluctuating trend of resistance but the prevalence remained above 70%. The first NSAR report in 2005 showed a national gentamicin resistance rate of 78.1% and this increased to more than 90% in 2007 and 2008 before dropping to around 80% in 2009. Individual studies of isolates obtained within that period showed contrasting results. The prevalence of gentamicin resistance in 66 MRSA isolates collected from an unnamed teaching hospital in 2003, 2004, and 2007 was 76% [16]. In another study, 32 isolates collected in 2006 and 2007 from a likewise undetermined hospital showed a similar resistance rate of 78.1% [17]. Isolates from HUSM, which is located in the north-eastern state of Kelantan in Peninsular Malaysia, appeared to display high prevalence of gentamicin resistance in the 2000s. The 1979 HUSM isolates collected from 2002 to 2007 showed a gentamicin resistance rate of 92.0% [15] and a 100% gentamicin resistance rate for 34 isolates obtained in 2008 [19]. However, no further reports of *S. aureus* isolates from HUSM were available in the 2010s. From 2010 onwards, data obtained from the NSAR reports showed a steady decline in the national prevalence of gentamicin resistance among Malaysian MRSA isolates. Between 2010 and 2012, gentamicin resistance was recorded in the range of 70% to 77% [28,29,30] and decreased to 62.5% and 46.9% in 2013 and 2014, respectively [31,32]. The latest NSAR report showed that gentamicin resistance among MRSA isolates has dropped further to only 11.2% in 2017 [5] (Figure 1). 

The prevalence of gentamicin resistance among MSSA isolates were much lower, with NSAR reporting rates of below 3%, except for the year 2011 and 2014 where the resistance was recorded at 3.6% and 3.0%, respectively [29,32]. The HUSM study of 124 MSSA isolates obtained in 2008 indicated a gentamicin resistance rate of 8.0% [19]. However, NSAR did not mention the prevalence of antimicrobial resistance for MSSA isolates in their reports from 2015 onwards.

**Figure 2:**
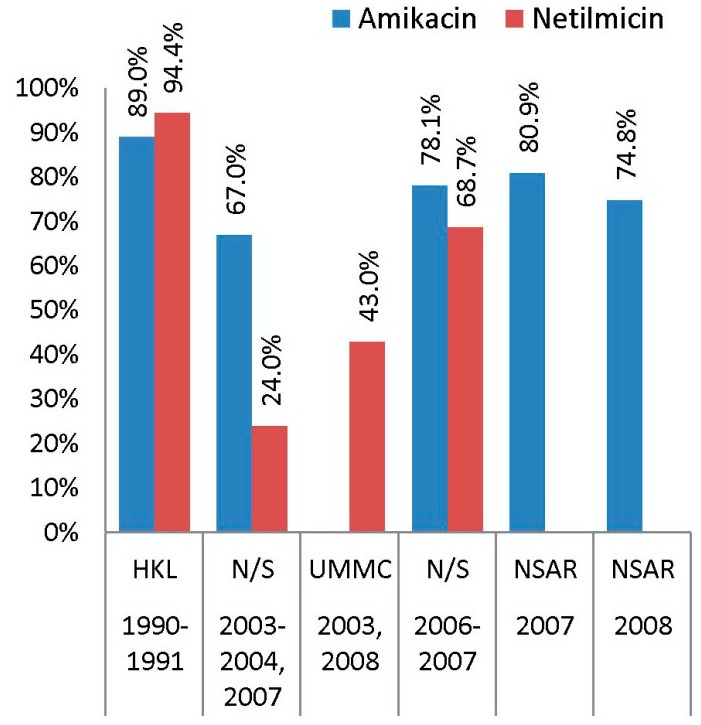
The prevalence of amikacin and netilmicin resistance among Malaysian MRSA isolates, 1990–2008. Data from the NSAR reports; HKL between 1990 and 1991 [13]; not specified (N/S) between 2003 and 2004 and in 2007 [16], and between 2006 and 2007 [17]; and UMMC in 2003 and 2008 [18].

Data on amikacin and netilmicin resistance among Malaysian *S. aureus* isolates were only available for MRSA isolates. However, data from two NSAR reports in 2007 and 2008 included the amikacin resistance rate for all *S. aureus* isolates (both MRSA and MSSA), which were 78.0% and 65.0%, respectively [25,26]. Amikacin resistance among MRSA isolates is generally in the upper range, with resistance rates mostly reported above 70% (Figure 2). However, one study on 66 MRSA isolates from a local teaching hospital in 2003, 2004, and 2007 reported amikacin resistance at a slightly lower range of 67.0% [16], whereas our more recent study involving 90 MRSA isolates obtained from HSNZ in 2016 and 2017 recorded an even lower rate, which was 12.2% [23].

Netilmicin resistance among MRSA isolates had a more fluctuating pattern (Figure 2), with the lowest prevalence of resistance, reported at 24.0% by Thong et al., for isolates obtained in 2003, 2004, and 2007 [16], while the highest prevalence of resistance was reported among 539 HKL MRSA isolates collected in 1990 and 1991, which was 94.4% [13]. Neela et al. also tested their 32 MRSA isolates collected in 2006 and 2007 for susceptibilities towards amikacin, netilmicin, and kanamycin, besides gentamicin. The aminoglycoside resistance rates for their MRSA isolates were similar (both to gentamicin and amikacin at 78.1% and kanamycin at 75.0%), with netilmicin the lowest at 68.7% [17].

### 2.3. Macrolides (Erythromycin)

**Figure 3:**
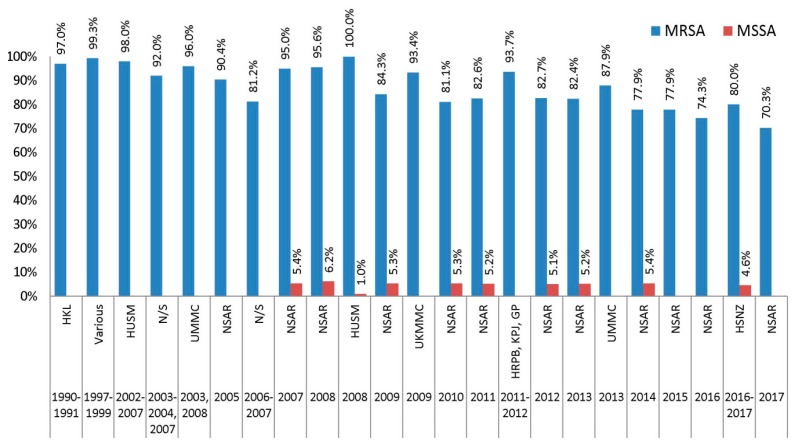
The prevalence of erythromycin resistance among Malaysian *S. aureus* isolates, 1990–2017. Data from the NSAR reports; HKL between 1990 and 1991 [13]; Various, collected from ten hospitals throughout Malaysia between 1997 and 1999 [14]; HUSM between 2002 and 2007 [15] and in 2008 [19]; not specified (N/S) between 2003 and 2004 and in 2007 [16], and between 2006 and 2007 [17]; UMMC in 2003 and 2008 [18], and in 2013 [22]; UKMMC in 2009 [20]; HRPB, KPJ and GP between 2011 and 2012 [21]; and HSNZ between 2016 and 2017 [23].

Almost all Malaysian MRSA isolates in the 1990s and early 2000s were erythromycin resistant with resistance rates of >90% (Figure 3). The first NSAR report in 2005 showed a national prevalence of 90.5% in 2005 and this increased to 95% in 2007 and 2008. Other studies of isolates obtained around this period also indicated very high prevalence of erythromycin resistance. Only a study on 32 MRSA isolates collected in 2006 and 2007 from an undisclosed tertiary hospital revealed a lower erythromycin resistance rate of 84.3% [17]. However, the NSAR report in 2009 showed that the prevalence of erythromycin resistance among the Malaysian MRSA isolates had decreased to 84.3% [27] and remained around the 80% level until 2013. The national resistance rates for erythromycin decreased further to 70.3% in the latest NSAR report of 2017 [5]. 

As for MSSA isolates, data from NSAR were only available from 2007 until 2014, where the resistance rates stayed consistent at around 5% to 6% (Figure 3). Only two independent studies reported erythromycin resistance among MSSA isolates, where the rate was much lower compared to the national rates with only approximately 1.0% among 124 MSSA isolates collected in HUSM in 2008 [19], whereas the HSNZ MSSA isolates recorded a slightly higher rate at 4.6%. 

### 2.4. Lincosamides (Clindamycin)

**Figure 4:**
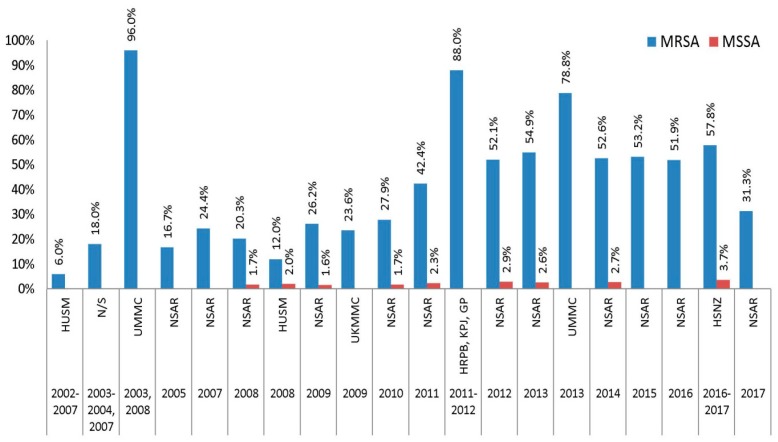
The prevalence of clindamycin resistance in Malaysian *S. aureus* isolates, 2002–2017. Data from the NSAR reports; HUSM between 2002 and 2007 [15] and in 2008 [19]; not specified (N/S) between 2003 and 2004 and in 2007 [16], and between 2006 and 2007 [17]; UMMC in 2003 and 2008 [18], and in 2013 [22]; UKMMC in 2009 [20]; HRPB, KPJ and GP between 2011 and 2012 [21]; and HSNZ between 2016 and 2017 [23].

From 2002 until 2010, data from the NSAR reports as well as various studies showed that the prevalence of clindamycin resistance among Malaysian MRSA isolates is mostly low, with resistance rates hardly reaching 30% (Figure 4). The lowest prevalence of resistance was recorded at 6.0% in 1979 HUSM MRSA isolates collected from 2002 to 2007 [15]. A subsequent study of 34 HUSM MRSA isolates collected in 2008 showed an increase in the prevalence of clindamycin resistance to 12% [19]. However, a study of 162 MRSA isolates from UMMC collected in 2003 and 2008 yielded an extremely high prevalence of clindamycin resistance at 96.0% [18]. The NSAR reports from that time period showed a prevalence of between 20%–30%. Nevertheless, in 2011 there was an increase in the national clindamycin resistance rate to 42.4% and this increased further to 52.1% in 2012. One study reported a very high resistance rate of 88.0% among MRSA isolates obtained from HRPB, KPJ, and GP in Ipoh [21]. The national clindamycin resistance rate maintained around the 50–55% level from 2013 to 2016 until it suddenly decreased by nearly 20% to 31.3% in the 2017 NSAR report [5]. During this time period, one study reported a resistance rate of 78.0% among isolates obtained from UMMC in 2013, although this could be attributed to the lower number of samples studied, which was 67 [22].

Data concerning clindamycin resistance among national MSSA isolates were only available from 2008 until 2014, where the resistance rates remained low throughout the years, i.e., below 3.0%, with the exception for our recent study of 109 HSNZ MSSA isolates from 2016 and 2017, where the prevalence of clindamycin resistance was slightly higher at 3.7% [23] (Figure 4).

### 2.5. Streptogramins (Quinupristin-Dalfopristin)

There is scarce data on the prevalence of quinupristin-dalfopristin resistance among Malaysian *S. aureus* isolates. Neela et al. reported that all 32 MRSA isolates from a tertiary hospital in 2006 and 2007 were fully susceptible to quinupristin-dalfopristin. We also recorded a similar observation in the MRSA and MSSA isolates obtained from HSNZ in 2016 and 2017 [23]. The only available data from NSAR regarding resistance to this antimicrobial was in year 2011, in which resistance was recorded as 3.9% for all *S. aureus* isolates (i.e., both MRSA and MSSA) [29].

### 2.6. Macrolide-Lincosamide-Streptogramin B (MLS_B_) Phenotypes

As a result of overlapping binding sites for macrolides, lincosamides, and streptogramin B, resistance involving ribosomal modification will affect the binding affinity of all three drugs. This is the situation observed in macrolide-lincosamide-streptogramin B (MLS_B_) resistance, where the resistance mechanism lies in the production of an enzyme called erythromycin ribosome methylase, encoded by the *erm* gene, which causes ribosomal modification by catalyzing the dimethylation of nucleotide 2058A [35]. The MLS_B_ resistance can be categorized as constitutive (cMLS_B_), inducible (iMLS_B_), or macrolides streptogramin (MS) phenotype. Out of these three, the iMLS_B_ phenotype is of utmost importance in the clinical settings. In this phenotype, the isolate appears to be erythromycin resistant and clindamycin susceptible in normal disc diffusion assays but the use of clindamycin as antimicrobial therapy in patients that were infected with iMLS_B_ strains has been associated with treatment failure [36]. The iMLS_B_ (or inducible clindamycin resistance) phenotype could only be detected through double-disc diffusion or D-zone test where erythromycin (15 µg) and clindamycin (2 µg) discs were placed 15 mm apart and iMLS_B_ isolates would appear resistant to erythromycin and have a clindamycin zone of ≥21 mm with a D-shaped zone [10].

There was limited data available for the prevalence of inducible clindamycin resistance among Malaysian *S. aureus* isolates. Earlier studies reported a very high prevalence of the iMLS_B_ phenotype. Neela et al. reported an iMLS_B_ prevalence of 96.1% among 32 MRSA isolates obtained in 2006 and 2007 [17]. Similarly, Lim et al. also found an iMLS_B_ prevalence of 96% among their 156 erythromycin-resistant MRSA isolates collected from UMMC in 2003 and 2008 [18]. Our more recent study however documented a much lower iMLS_B_ prevalence among HSNZ isolates obtained in 2016 and 2017, which was 46.7% among 90 MRSA isolates and 1.8% among 109 MSSA isolates [23].

### 2.7. Tetracyclines (Tetracycline, Doxycycline, and Minocycline)

**Figure 5:**
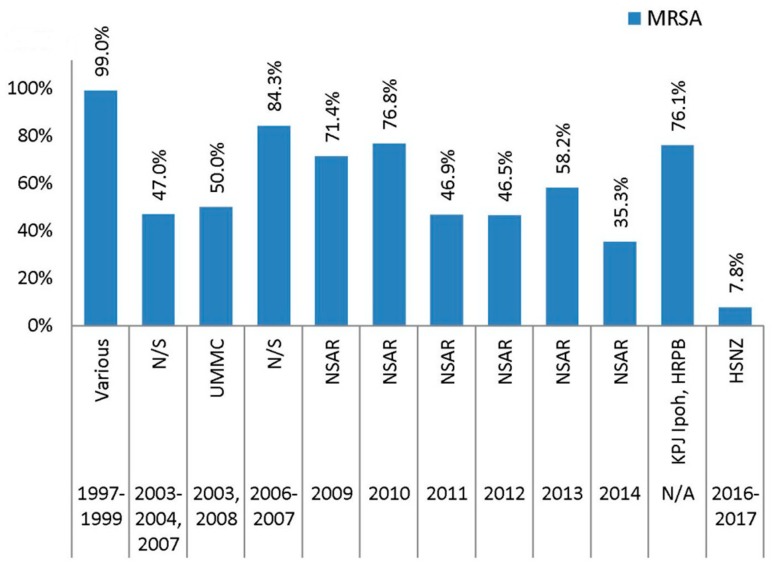
The prevalence of tetracycline resistance among Malaysian MRSA isolates, 1997–2017. Data from the NSAR reports; Various, collected from ten hospitals throughout Malaysia between 1997 and 1999 [14]; not specified (N/S) between 2003 and 2004 and in 2007 [16], and between 2006 and 2007 [17]; UMMC in 2003 and 2008 [18]; KPJ and HRPB with an unspecified year of collection [37]; and HSNZ between 2016 and 2017 [23].

The CDC-ECDC proposed guidelines listed three agents in the tetracycline class of antimicrobials, namely tetracycline, doxycycline, and minocycline, for *S. aureus* susceptibility testing [11]. NSAR data for the Malaysian MRSA isolates only covered the prevalence of tetracycline resistance and only for the period of 2009–2014. Focusing just on the NSAR data, the prevalence of tetracycline resistance in the Malaysian MRSA isolates was at a downward trend with the highest prevalence at 76.8% in 2010 and the lowest at 35.3% in 2014. An early report of 400 MRSA isolates obtained from ten hospital laboratories throughout Malaysia between 1997 and 1999 [14] (Figure 5) showed nearly all isolates (99.0%) were resistant to tetracycline. From 2003 to 2008, data from two separate studies showed that the prevalence of tetracycline resistance was around 50% [16,18], but another study on MRSA isolates obtained in 2006 and 2007 showed a higher prevalence of 84.3% [17]. A recent study documented a similarly high rate of tetracycline resistance (76.1%) among 117 MRSA isolates obtained from HRPB and KPJ in Ipoh [37]. However, the study did not mention the year of the sample collection. The lowest tetracycline resistance was reported at 7.8%, involving 90 MRSA isolates obtained from HSNZ in 2016 and 2017 [23]. Thus, over a period of nearly 30 years, the prevalence of tetracycline resistance appeared to decrease in MRSA isolates from Malaysia. 

So far, only one study has reported on the prevalence of minocycline resistance among 32 MRSA isolates obtained in 2006 and 2007 and with a rate of 65.6% [17], while doxycycline resistance has yet to be documented.

### 2.8. Fluoroquinolones (Ciprofloxacin, Moxifloxacin, Norfloxacin, and Ofloxacin)

**Figure 6:**
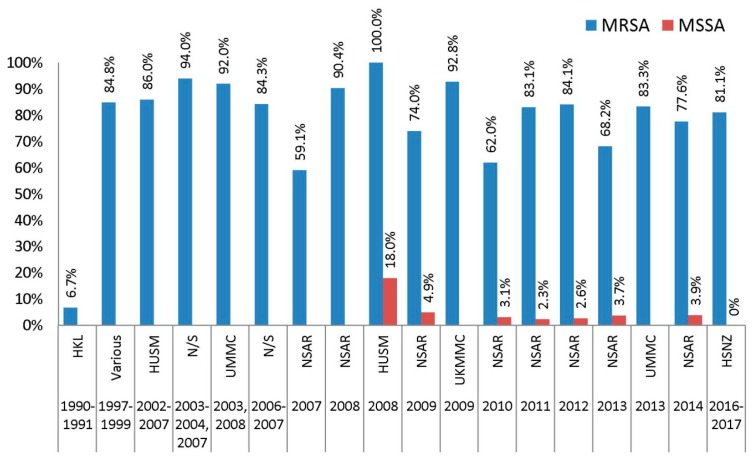
The prevalence of ciprofloxacin resistance among Malaysian *S. aureus* isolates, 1990–2017. Data from the NSAR reports; HKL between 1990 and 1991 [13]; Various, collected from ten hospitals throughout Malaysia between 1997 and 1999 [14]; HUSM between 2002 and 2007 [15] and in 2008 [19]; not specified (N/S) between 2003 and 2004 and in 2007 [16], and between 2006 and 2007 [17]; UMMC in 2003 and 2008 [18], and 2013 [22]; UKMMC in 2009 [20]; and HSNZ in 2016 and 2017 [23].

The earliest report on ciprofloxacin resistance in Malaysian MRSA isolates was on 539 MRSA isolates from HKL that were obtained in 1990 and 1991 [13], whereby a very low prevalence of 6.7% was reported (Figure 6). A subsequent report on isolates obtained from ten different hospitals in 1997–1999 showed a dramatically higher prevalence of 84.8% [14]. MRSA isolates from various studies obtained in 2002–2009 showed a consistently high prevalence of ciprofloxacin resistance (>85%) [14,15,16,22,23]. However, the first NSAR report indicated a national resistance rate of 59.1% for 2007, but this rate increased substantially to 90.4% the following year. The national ciprofloxacin resistance rates fluctuated between 62–84% from 2009 until the final NSAR report for ciprofloxacin resistance on 2014. Our recent report on MRSA isolates from HSNZ in 2016–2017 still indicated a high prevalence of ciprofloxacin resistance, at 81.1% [23]. 

MSSA isolates were mostly susceptible to ciprofloxacin, with NSAR reports from 2009–2014 indicating resistance rates of between 2–5%. However, Al-Talib et al. found a higher rate of ciprofloxacin resistance (18.0%) among their 124 HUSM MSSA isolates collected in 2008 [19], whereas our recent data on HSNZ isolates in 2016 and 2017 showed that all MSSA isolates were susceptible to ciprofloxacin [23]. 

Data on resistance towards other fluoroquinolone antimicrobials among Malaysian *S. aureus* isolates were scarce (Figure 6). An early study reported a 3% prevalence of resistance towards moxifloxacin among 32 MRSA isolates which were obtained from nine major hospitals between 1997 and 1999 [38]. However in this study, the authors only chose to test isolates that were both resistant to fusidic acid and rifampicin, hence the low number of isolates may not truly reflect the prevalence of moxifloxacin resistance among MRSA isolates for that particular period [38]. Neela et al. tested their 32 MRSA isolates obtained in 2006 and 2007 against two other fluoroquinolone antimicrobials, which were norfloxacin and ofloxacin, and found the prevalence of resistance towards both agents was at 78.1% and 53.1%, respectively [17]. 

### 2.9. Folate Pathway Inhibitors (Co-Trimoxazole)

**Figure 7:**
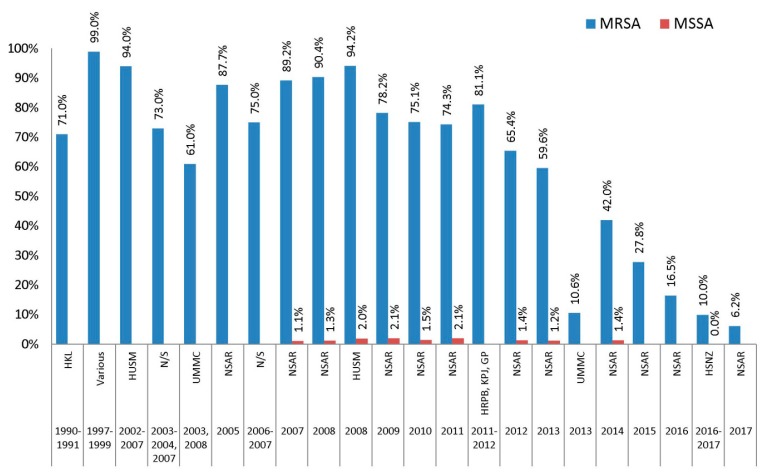
Prevalence of co-trimoxazole resistance in Malaysian *S. aureus* isolates, 1990–2017. Data from the NSAR reports; HKL between 1990 and 1991 [13]; various, collected from ten hospitals throughout Malaysia between 1997 and 1999 [14]; HUSM between 2002 and 2007 [15] and in 2008 [19]; not specified (N/S) between 2003 and 2004 and in 2007 [16], and between 2006 and 2007 [17]; UMMC in 2003 and 2008 [18], and in 2013 [22]; UKMMC in 2009 [20]; HRPB, KPJ and GP between 2011 and 2012 [21]; and HSNZ between 2016 and 2017 [23].

There was generally a high prevalence (>70%) of co-trimoxazole resistance in the Malaysian MRSA isolates from the 1990s to 2000s, with fluctuations in between (Figure 7). The NSAR reports for 2005–2008 documented a resistance prevalence of close to 90%, but which gradually declined to around 70% by 2011. Studies in HUSM supported this as MRSA isolates from 2002–2007 showed prevalence of co-trimoxazole resistance at 94.0% [15] and 94.2% for isolates from 2008 [19]. Studies from other healthcare settings, however, showed a lower prevalence of co-trimoxazole, with the lowest rate at 61.0% in UMMC isolates collected in 2003 and 2008 [18]. Interestingly, the national prevalence of co-trimoxazole resistance started to noticeably decline from 2012 onwards, with rates of 65.4% in 2012 to a very low level of 6.2% in 2017. Studies from individual hospitals showed support for this trend with MRSA isolates obtained from UMMC in 2013–2014 indicating a prevalence of 10.6% [22], whereas our recent study of HSNZ MRSA isolates from 2016–2017 also showed a similar prevalence of 10.0% [23]. The reason for this drastic decline in co-trimoxazole resistance in the Malaysian MRSA isolates is not known as we do not have data for the use of co-trimoxazole and other antibiotics in the treatment of MRSA and other bacterial infections in the Malaysian hospitals. Most Malaysian MSSA isolates were still very much susceptible to co-trimoxazole, where data recorded from 2007 until 2014 showed the resistance rates lingering around 1.1% to 2.1% (Figure 7).

### 2.10. Phenicols (Chloramphenicol)

**Figure 8:**
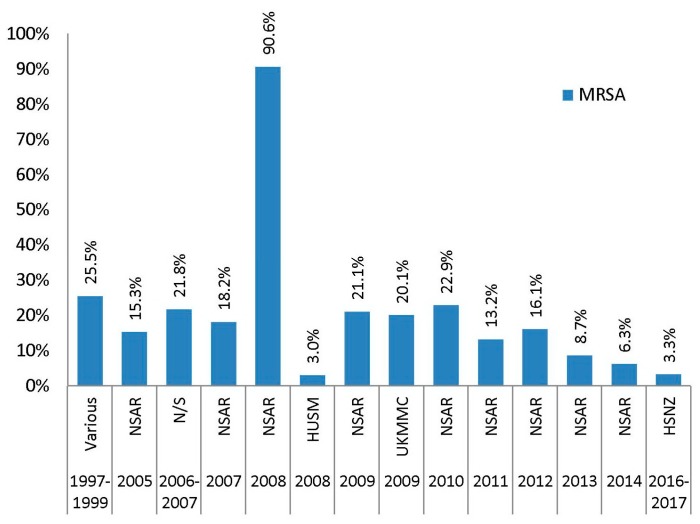
The prevalence of chloramphenicol resistances in Malaysian MRSA isolates, 1997–2017. Data from the NSAR reports; various, collected from ten hospitals throughout Malaysia between 1997 and 1999 [14]; not specified (N/S) between 2006 and 2007 [17]; HUSM in 2008 [19]; UKMMC in 2009 [20]; and HSNZ in 2016 and 2017 [23].

Malaysian MRSA isolates were moderately resistant to chloramphenicol. In most NSAR reports as well as individual studies, the prevalence of chloramphenicol resistance was observed below 30% (Figure 8). The NSAR report for 2008, however, documented a great increase in chloramphenicol resistance among MRSA isolates, with a documented rate of 90.6% [25]. This was the highest prevalence ever reported and was markedly different from the rest of the NSAR reports and studies, prompting doubt as to the accuracy of the 2008 NSAR data regarding chloramphenicol resistance. Data from 2009 until 2010 showed a prevalence of resistance in the range of 20% to 23%, but which gradually declined in 2012 (16.1%) until the final NSAR report for MRSA chloramphenicol resistance in 2014 (6.3%). Our recent report on HSNZ isolates from 2016–2017 supported this decline, where we observed prevalence of chloramphenicol resistance was only at 3.3% [23]. Data regarding chloramphenicol resistance among Malaysian MSSA isolates were not covered by the NSAR reports. Our recent study on 109 HSNZ MSSA isolates indicated their full susceptibility towards chloramphenicol [23].

### 2.11. Ansamycins (Rifampin)

**Figure 9:**
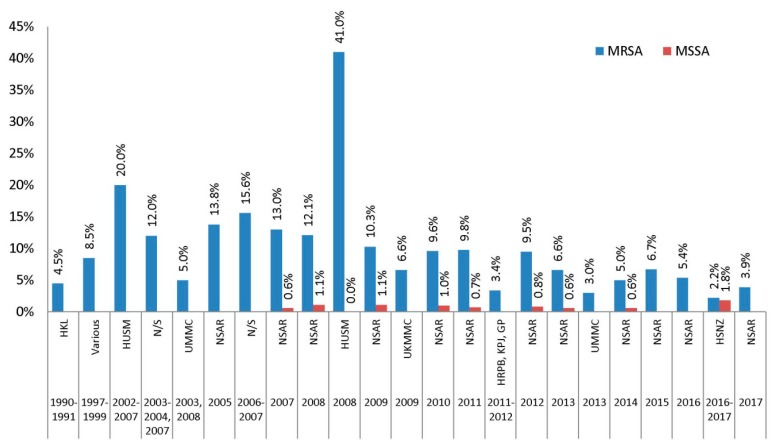
The prevalence of rifampin resistance in Malaysian *S. aureus* isolates, 1990–2017. Data from the NSAR reports; HKL between 1990 and 1991 [13]; various, collected from ten hospitals throughout Malaysia between 1997 and 1999 [14]; HUSM between 2002 and 2007 [15] and in 2008 [19]; not specified (N/S) between 2003 and 2004 and in 2007 [16], and between 2006 and 2007 [17]; UMMC in 2003 and 2008 [18], and in 2013 [22]; UKMMC in 2009 [20]; HRPB, KPJ and GP between 2011 and 2012 [21]; and HSNZ between 2016 and 2017 [23].

The prevalence of rifampin resistance in Malaysian MRSA isolates is generally low, mostly around 10–15% in the 2000s and dropping to below 10% in the 2010s, with the latest NSAR report of 2017 showing a prevalence of only 3.9% (Figure 9). However, the prevalence of rifampin resistance in MRSA isolates from two studies carried out in HUSM were much higher than the national average. HUSM isolates from 2002 to 2007 showed a resistance rate of 20% [15] and this increased considerably to 41% for isolates obtained in 2008 [19].

As for MSSA isolates, the prevalence of rifampin resistance was very low, as observed from data available from 2007 to 2014, with resistance rates rarely reaching 1% (Figure 9). We reported a slightly higher prevalence of rifampin resistance (1.8%) among the HSNZ MSSA isolates from 2016–2017 [23].

### 2.12. Glycopeptides (Vancomycin and Teicoplanin)

Resistance to vancomycin can exist in three forms depending on the minimum inhibition concentration (MIC) values. Isolates showing intermediate resistance towards vancomycin (vancomycin-intermediate *S. aureus*, VISA) have MIC values of 4–8 µg/mL, while those with complete resistance have MIC values of ≥16 µg/mL [10]. Heterogeneous VISA (hVISA) isolates, on the other hand, have susceptible MIC values (≤2 µg/mL), but a proportion of the population also have MIC values in the intermediate range [39].

In the earliest NSAR reports, which were from years 2003 to 2005, the vancomycin resistance rate was recorded at 0.1% for three consecutive years [24]. This was the rate recorded among all *S. aureus* isolates, including MRSA. In 2005, NSAR reported a similar resistance rate, but exclusively for MRSA isolates [24]. Not much information could be obtained from these early reports. The numbers as well as the name of participating hospitals were not listed and further information regarding these vancomycin-resistant isolates such as the origin, source of the isolates, and how the vancomycin resistance was tested (whether by disc diffusion method or MIC value) were also not available. The validity of these results could also not be confirmed as there were no peer-reviewed published reports regarding these supposedly vancomycin-resistant isolates. However, in the following years, there have been no reports of vancomycin-resistant isolates in any of the NSAR reports as well as in any individual studies. 

While complete resistance towards vancomycin has not been reported in Malaysia in published journals, several studies have documented the presence of VISA and hVISA isolates. The hVISA isolates were mostly detected among isolates collected in 2009. Seven out of 320 MRSA isolates collected from HKL in 2009 were hVISA isolates [40], while two out of 43 MRSA isolates collected during the same year from Hospital Selayang were also confirmed as hVISA. A report of the emergence of a VISA isolate in Malaysia was just made recently, where the isolate was obtained from a female patient who was first admitted for leptospirosis in an unnamed referral hospital. The isolate showed a vancomycin MIC value of 4 µg/mL and was obtained from the blood sample of the patient who had previously received a total of 31 days of intravenous vancomycin therapy during her hospital stay [41].

Teicoplanin resistances were only reported in the 2007 and 2008 NSAR, where the rate was recorded at 0.2% for MRSA isolates in 2007 [26]. In 2008, the rate was recorded at 0.1% for MRSA isolates and 0.2% for MSSA isolates [25]. However, it should be noted that these rates were marked as unconfirmed in the NSAR reports. In a study involving 162 MRSA isolates obtained from UMMC in 2003 and 2008, teicoplanin resistance was recorded at 1%, which was just slightly higher than the national rate [18]. In another study, 32 MRSA isolates collected from an unnamed tertiary hospital from 2006 to 2007 showed full susceptibility towards teicoplanin [17]. A similar observation was also reported among 124 MSSA and 34 MRSA isolates obtained in 2008 from HUSM, where all the isolates showed full sensitivity towards teicoplanin [19]. Teicoplanin-resistant isolates were also not detected among the 318 MRSA isolates collected in Universiti Kebangsaan Malaysia Medical Centre (UKMMC) in 2009 [20].

### 2.13. Oxazolidinones (Linezolid)

**Figure 10:**
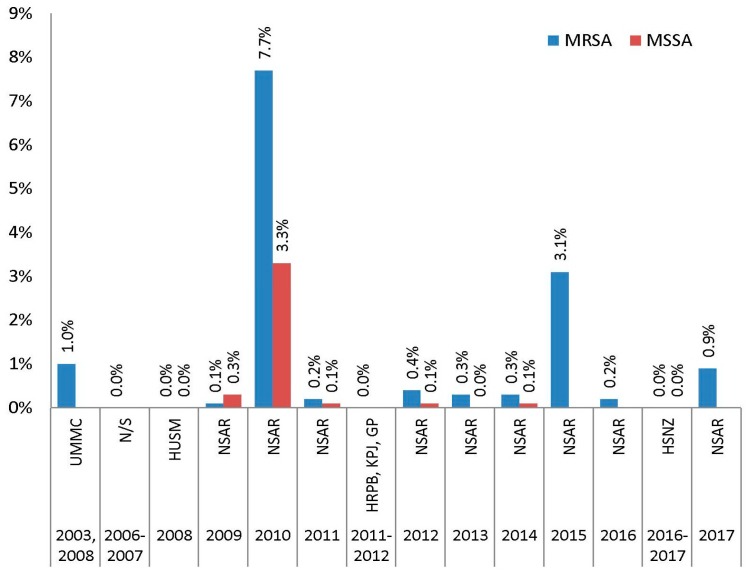
The prevalence of linezolid resistance in Malaysian *S. aureus* isolates, 2003–2017. Data from the NSAR; UMMC in 2003 and 2008 [18]; not specified (N/S) between 2006 and 2007 [17]; HUSM in 2008 [19]; HRPB, KPJ and GP between 2011 and 2012 [21]; and HSNZ in 2016 and 2017 [23].

The prevalence of linezolid resistance in Malaysian *S. aureus* isolates was very low, with most NSAR data and other individual studies reporting resistance rates below 1.0% (Figure 10). However, NSAR reports for 2010 and 2015 documented higher linezolid resistance rates with 7.7% for MRSA and 3.3% for MSSA isolates in 2010, and 3.1% for MRSA isolates in 2015 (MSSA data was unavailable for 2015). The latest NSAR report for 2017 showed a linezolid resistance rate of 0.9% for MRSA. 

### 2.14. Fucidanes (Fusidic Acid)

**Figure 11:**
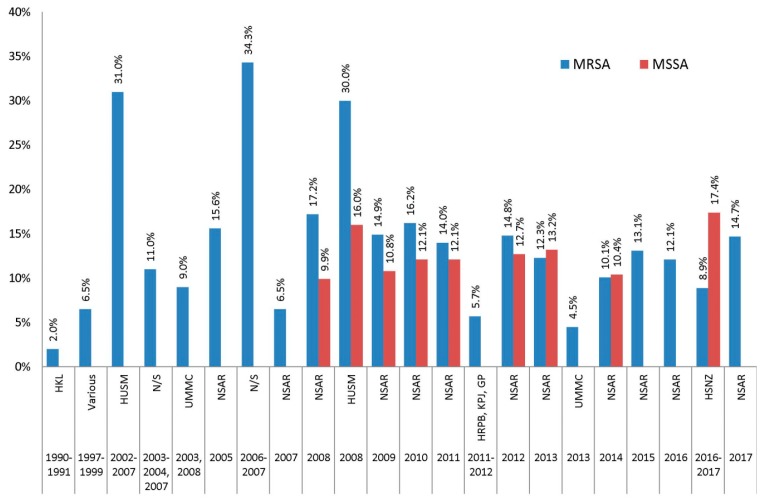
The prevalence of fusidic acid resistance in Malaysian *S. aureus* isolates, 1990–2017. Data from the NSAR reports; HKL between 1990 and 1991 [13]; various, collected from ten hospitals throughout Malaysia between 1997 and 1999 [14]; HUSM between 2002 and 2007 [15] and in 2008 [19]; not specified (N/S) between 2003 and 2004 and in 2007 [16], and between 2006 and 2007 [17]; UMMC in 2003 and 2008 [18], and in 2013 [22]; UKMMC in 2009 [20]; HRPB, KPJ and GP between 2011 and 2012 [21]; and HSNZ between 2016 and 2017 [23].

The NSAR reports indicated that the prevalence of fusidic acid resistance in Malaysian MRSA isolates remained relatively consistent between 10–16% from the first NSAR report in 2005 (15.6%) to the latest report in 2017 (14.7%), apart from 2007 when NSAR reported a prevalence of only 6.5% (Figure 11). Individual studies, particularly from HUSM, showed much higher prevalence of fusidic acid resistance in their MRSA isolates, with 31.0% for isolates collected from 2002–2007 [15] and 30.0% for isolates obtained in 2008 [19]. MRSA isolates obtained from an unspecified healthcare centre in 2006–2007 also showed higher prevalence of fusidic acid resistance at 34.3% [17]. From 2011 onwards, fusidic acid resistance isolates were reported in three independent studies, with HSNZ isolates showing higher resistance rate among MSSA isolates (17.4%) compared to MRSA isolates (8.9%) [21,22,23].

### 2.15. Glycylcyclines (Tigecycline)

There was very limited data concerning tigecycline resistance among Malaysian *S. aureus* isolates. NSAR did not report the prevalence of tigecycline resistance and individual studies were also very scarce. One such study was by Al-Talib et al., who found full tigecycline susceptibility among the HUSM MRSA and MSSA isolates obtained in 2008 [19]. Isolates collected approximately a year later (2009–2010) in HKL showed a higher rate of resistance to tigecycline among MRSA isolates upon determination of MIC values, which was 26.7% [42]. Both studies involved a small number of MRSA isolates (34 isolates in the first study and 30 isolates in the latter) and yet there was a difference in the resistance rates between the two studies. A more recent study, however, documented a much lower prevalence of tigecycline resistance at 5.6% among 90 HSNZ MRSA isolates in 2016 and 2017 [23]. Tigecycline is a useful alternative for the treatment of vancomycin-resistant isolates. Therefore, the rise in tigecycline resistance should not be taken lightly and overuse of this antibiotic should be avoided.

### 2.16. Lipopeptides (Daptomycin)

Daptomycin is a relatively new antimicrobial, having been approved for clinical use in 2003. Reports on daptomycin resistance among Malaysian *S. aureus* isolates remain very scarce. The national resistance rate for daptomycin was not available in any of the NSAR reports. To the best of our knowledge, there has only been one study involving 30 MSSA and 30 MRSA isolates collected from HKL during 2009 and 2010, where a 100% susceptibility rate for daptomycin was recorded when tested using E-test strips to obtain the MIC values [42].

### 2.17. Overview of Staphylococcus aureus Antimicrobial Resistance Trends in Malaysia

Generally, the resistance rates reported by NSAR seemed to be lower than that of individual hospitals, possibly due to the pooling effect. NSAR reported the compilation of antibiotic susceptibility data from hospital microbiology laboratories mainly of government hospitals and university hospitals from all the 13 states in Malaysia. Hence, the sample size of each NSAR report was frequently above 10,000 isolates, which is more than 10 times higher than the sample sizes of individual reports from selected hospitals (which were well below a thousand).

Amikacin and netilmicin, two older line of drugs that were previously used were stopped for empirical treatment of MRSA. According to the second edition of the Malaysian National Antibiotic Guideline 2014, drugs to be used for treating MRSA infections include clindamycin, erythromycin, vancomycin, rifampicin, tetracycline, linezolid, and gentamicin [43]. The second edition of the National Antibiotic Guideline 2014 is in line with the Protocol on Antimicrobial Stewardship (AMS) Program in healthcare facilities launched at the end of 2014 [44]. Overall, the prevalence of resistance to some of these drugs seems to be reduced as the positive outcome of the implementation of the AMS program [45]. The five core strategies of AMS are as follows: (i) Formulation of an AMS team in each hospital, health district office and health clinics; (ii) surveillance and feedback mechanism on specific antimicrobial consumption; (iii) implementation of prospective audit and feedback according to local needs; (iv) formalize regular antimicrobial rounds by AMS team especially in state and specialist hospitals; and (v) establishment of formulary restriction and a pre-authorization/approval system [46].

The decreasing resistance to the older line of drugs observed in this review for Malaysia has also been described in the United States and China [47,48]. Possible explanations for this scenario include a more rational use of antibiotics and a shift in antimicrobial selection pressures [47,48]. In Malaysia, vancomycin or linezolid are the drug of choice to treat MRSA infections, whereas cloxacillin is the recommended antimicrobial therapy for MSSA infections, particularly for adult patients in the intensive care units [49]. These could thus result in less usage of older antimicrobials and therefore a shift in antibiotic pressures, ultimately leading to decrease resistance to the older drugs. Some authors also suggested the changing molecular epidemiology of *S. aureus* clones circulating in the hospital as a possible reason for the shift in antimicrobial resistance patterns [50,51].

## 3. Molecular Mechanisms of Antimicrobial Resistance

Data regarding the molecular mechanisms of antimicrobial resistance in Malaysia were limited. There were two studies describing the prevalence of tetracycline resistance genes among Malaysian MRSA strains, where *tetM* and *tetK* were found to be the most prevalent among tetracycline-resistant MRSA isolates. Ong et al. (2017) found that *tetM* was the predominant gene, with 97.8% prevalence compared to 42.7% for *tetK*, among 89 tetracycline-resistant MRSA isolates and also with a 90.0% prevalence of *tetM* among 10 MRSA isolates that were intermediately-resistant [37]. Similarly, in another study, *tetM* was also found to be the more predominant tetracycline-resistant gene, with a prevalence of 49.0% compared to *tetK* (21.0%), among 188 MRSA isolates [52]. The higher prevalence of *tetM* was postulated to be due to the stable nature of Tn*916*, the *tetM*-containing conjugative transposon [37,53], whereas *tetK* is usually carried in a small plasmid (pT181) and therefore can only affect a smaller number of hosts compared to Tn*916*, resulting in a lower prevalence [37,54].

The prevalence of MLS_B_ resistance genes among clinical MRSA isolates has only been described in one clinical study. Lim et al. (2012) reported a prevalence of 84.0% for *ermA*, 21.0% for *ermC*, and only 2.0% for *msrA* among 188 MRSA hospital isolates [52]. In a community study, 49 *S. aureus* isolates obtained from 200 healthy undergraduate students in 2012 and 2013 yielded eight erythromycin-resistant isolates, of which six were found to carry the *msrA* gene and one isolate harbored *ermC* [55].

In *S. aureus*, the multidrug efflux pump NorA (encoded by the *norA* gene) provides a low level resistance to fluoroquinolone antibiotics [56,57], whereas the efflux pump MdeA (encoded by *mdeA* gene) can confer resistance to fusidic acid [58]. The prevalence of these efflux genes in 16 MRSA/MSSA isolates from HUKM was reported, whereby the *norA* gene was detected in 13 isolates whereas the *mdeA* gene was detected in 15 isolates [59].

## 4. Clonal Types and Characteristics of Malaysian *S. aureus* Isolates

MRSA isolates can be differentiated into two categories depending on the origin of the isolates, i.e., hospital-associated MRSA (HA-MRSA) and community-associated MRSA (CA-MRSA). As the name implies, HA-MRSA is typically found in the hospital settings whereas CA-MRSA isolates originate from the community. The CDC defines CA-MRSA as an isolate obtained within 48 hours after hospitalization with no previous history of hospitalization, surgery, residence in long-term care facility, and dialysis within the last 12 months, presence of percutaneous device or indwelling catheter, and previous MRSA infection or colonization. Isolates that do not fulfill the above definition are considered HA-MRSA [60]. There are several distinguishing features between the two types of MRSA. While HA-MRSA generally affects people with chronic diseases, hospitalized patients and the elderly, CA-MRSA usually affects young and healthy individuals [61]. Clinically, most CA-MRSA infections involve the skin and soft tissue, while HA-MRSA has a wider spectrum of disease [62]. Genetically, MRSA strains are commonly grouped into different clones based on their sequence types (STs) as well as staphylococcal cassette chromosome *mec* (SCC*mec*) types. Generally, HA-MRSA tend to carry SCC*mec* types I, II, and III while CA-MRSA tend to carry SCC*mec* types IV or V [63]. Identifying MRSA clones enables researchers to carry out surveillance and outbreak investigations. Some of the most well-known MRSA clones include the Brazillian clone (ST239-III), the Iberian clone (ST247-IA), the Berlin clone (ST45-IV), the New York/Japan clone (ST5-II), epidemic MRSA (EMRSA)-15 (ST22-IV-PVL negative), and EMRSA-16 (ST36-II) [64,65,66].

Various studies found that the predominant HA-MRSA clone circulating in Malaysian hospitals is ST239 belonging to clonal complex (CC) 8 and *spa* type t037, with the most common SCC*mec* type being type III or its variant IIIA. Ghaznavi-Rad et al. reported the majority of MRSA strains (92.8%) from HKL were ST239 belonging to CC8 for isolates collected from 2007–2008 [67]. These findings were similar to another study published in 2010, where 83.3% of their MRSA isolates were also found to be ST239 belonging to CC8 for isolates collected from 2006–2007 [68]. 

Other STs that have been documented in Malaysia include ST1, ST6, ST7, ST22, ST30 ST188, ST772, ST1178, and ST1283 [18,67,68,69,70,71] (Table 3). The emergence of ST22 is of particular interest as this strain mostly circulates in Europe and belongs to the EMRSA-15 [72]. ST22 is also a strain that is capable of quickly spreading and therefore able to replace the other strains [73]. The emergence of this strain means that it has spread to Malaysia and could replace the currently dominating ST239. In Singapore, MRSA ST239 containing SCC*mec* type III was the dominant clone in the mid-1980s, but was replaced by ST22-SCC*mec* type IV isolates beginning in the early 2000s [72]. However, from 2007 onwards, the genetic diversity of circulating ST239 clones showed an increase along with the emergence of a subclone of ST239, which was attributed to the multiple acquisition of the Arginine Catabolic Mobile Element (ACME) that encode genes associated with enhanced skin colonization [72]. 

Generally, SCC*mec* type III or its variant IIIA is the predominant type in Asian countries like Singapore, Indonesia, and Thailand [93]. This is also true for Malaysia, as demonstrated by Ghaznavi-Rad et al., where they documented 20% of SCC*mec* type III and 72.8% of SCC*mec* type IIIA in their isolates [67]. Other studies corroborating this finding include Neela et al., with 41.6% of type III and 52.7% of type IIIA [68], and Mustafa et al., who reported 78.5% of type III in their isolates collected from Hospital Tengku Ampuan Afzan [79]. Two other studies in 2009 and 2011 also have similar results with the former study and reported 90% of type III and the latter detected the same type in 96.8% (608/628) MRSA isolates [18,69]. Likewise, more recent studies from UMMC of MRSA isolates obtained from 2011 to 2013 indicated the majority were ST239-SCC*mec* type III, followed by ST22-SCC*mec* type IV [22,80], a situation that was similar to that in Singapore before 2007 [72]. Another paper that reported on MRSA isolates obtained in the Malaysian state of Perak in 2011 and 2012 also showed the predominance of SCC*mec* type III, but with the emergence of SCC*mec* type IV among 175 non-repetitive isolates [21]. Although the predominant clone in Malaysia appeared to be ST239 with SCC*mec* type III, Samat et al. made an interesting discovery of the first ST239 containing an SCC*mec* type II [78]. The strain, which was isolated from a skin swab in an orthopedic clinic, was the first of its kind to be reported.

The prevalence rate of CA-MRSA is much lower compared to HA-MRSA. The first CA-MRSA isolate in Malaysia was reported by Shamsudin et al. when they screened 100 healthy students for nasal carriage. Out of the 100 nasal cultures, 26 were positive for *S. aureus*, of which three were identified as CA-MRSA based on their SCC*mec* types, which were IVa and V [94]. In clinical settings, Sam et al. found two CA-MRSA isolates when performing an analysis of cases from 2002 to 2007 [74], while Rashid et al. discovered five isolates throughout 2007 [95] and Ahmad et al. documented nine isolates from a total of 628 MRSA strains collected from 2006–2008 [69].

One feature attributable to CA-MRSA infections is that they usually involve the skin and soft tissues. Several studies in Malaysia supported this theory as their CA-MRSA isolates were obtained from patients with skin and soft tissue infections [74,95]. Chan et al. presented a case report of necrotizing fasciitis of the left lower limb in a healthy 20 year old male due to CA-MRSA, which started out as a soft tissue infection [96]. 

Aside from the type of infection, another characteristic found to be associated with CA-MRSA is the possession of Panton-Valentine leucocidin (PVL), which is a pore-forming toxin. Isolates of CA-MRSA in Malaysia were mostly positive for PVL [69,74,94,95]. However, there were also reports of PVL-negative strains detected in these isolates [69,94]. 

The dominant CA-MRSA strains in Malaysia were found to mostly belong to ST30. The two CA-MRSA samples from Sam et al. were of ST6 and ST30 [74]. Ahmad et al. found eight ST30 strains from nine CA-MRSA isolates, while the remaining one isolate was ST80 [69]. One of the three isolates from Shamsudin et al. was ST80, while the other two were not found to match any type in the multilocus sequence typing (MLST) database [94]. 

While HA-MRSA generally harbors SCC*mec* type I, II, and III, CA-MRSA is often associated with type IV and V [63]. Shamsudin et al. reported two isolates with SCC*mec* type V and one with type IVA [94], while Rashid et al. also found four isolates with type IV [95]. However, two studies found isolates that did not meet the definition of CA-MRSA and yet harbored SCC*mec* types related to CA-MRSA. Sam et al. described seven nosocomial isolates carrying SCC*mec* type IV, with three of them even testing positive for PVL, and four strains belong to ST6 and ST30 [74]. Similarly, Ahmad et al. detected SCC*mec* type IV in 11 nosocomial samples, with three being PVL-positive. Two samples were ST30 and five had no match in the database [69].

Studies reporting on the clonal characteristics of MSSA isolates were limited in number. Even so, two studies reporting on the STs of Malaysian MSSA isolates described an interesting finding, in which MSSA appeared to be very genetically diverse. In one study, a group of researchers screened 268 MSSA isolates obtained from both the clinical and community settings in 2008 where they managed to discover as many as 26 different types of STs [76] (Table 3). Similarly, in another study involving 35 MRSA and 21 MSSA isolates collected between 2008 and 2010, the MSSA isolates also showed more diverse STs compared to MRSA isolates where eight STs were detected among MSSA isolates as compared to only two STs among MRSA isolates [77].

In recent years, sequencing of the full genome of pathogens via whole-genome sequencing (WGS) has revolutionized the global epidemiological investigation in hospitals and communities including for *S. aureus* [97,98]. However, until now, no large-scale WGS of *S. aureus* for epidemiological investigations from Malaysia has been published, with only limited reports of one or several isolates. In Malaysia, a brief WGS report was found for a HA-MRSA ST239 clone isolated from a patient with septicemia. This isolate was resistant to oxacillin, ampicillin, cefuroxime, ceftriaxone, gentamicin, erythromycin, ciprofloxacin, and co-trimoxazole [99]. Hashim et al. (2018) also conducted WGS for a hospital-acquired VISA isolate with a vancomycin MIC of 4 µg/mL from a patient who was admitted for leptospiral infection and received an intravenous course of vancomycin for 31 days prior to the detection of the VISA isolate [41]. They reported mutations in the *graR*, *graS*, *walR*, and *vraR* genes [41], but without further detailed genome analyses of the VISA isolate. Other WGS studies included one for a SCC*mec* V-ST772-PVL positive MRSA obtained from a blood sample of a newborn [70], a ST1-PVL positive MSSA obtained from pus [71], and three SCC*mec* III-ST239 and one SCC*mec* IV-ST1178 MRSA isolates [100]. All of these are brief reports without detailed analyses of the genome characteristics and phylogeny.

The importance of *S. aureus* as a pathogen not only lies in human populations, but also in animals and from the environment. This is fundamental for the One Health approach, which recognizes that human, animal, and environmental health are all interlinked [101]. The antibiotics used in livestock animals belong to the same group of those used for humans and the issue of antimicrobial resistance in livestock-associated MRSA (LA-MRSA) should be treated with equal concern as per a One Health problem. Irrational antibiotic use in animals has been shown to contribute to the rise of antimicrobial resistance where these resistant strains were also capable of affecting humans [102]. For instance, LA-MRSA has been shown to be capable of causing human infections [103]. In Malaysia, several studies have described the varying prevalence of *S. aureus* in different types of animals (Table 3), with the highest prevalence for *S. aureus* colonization at 44.0% (22/50) from the nares of horses [88] and the highest prevalence for MRSA was 18.0% (9/50) from the feathers of chickens [85]. One matter of concern is that some of these studies demonstrated high level of resistance among the *S. aureus* isolates. For example, one MRSA strain isolated from a cat was shown to have a high level of oxacillin resistance with an MIC of 256 µg/mL [87]. In another study, 13 MSSA isolates from horses were found to be multidrug resistant, with resistances to three to 10 types of antimicrobials [88]. This indicates the importance of continuous *S. aureus* surveillance among animals, as well as proper antimicrobial use, to minimize their contribution to increasing prevalence of antimicrobial resistance.

Thus far, data on antimicrobial usage in livestock in Malaysia is very limited. Neela et al. (2009) reported the detection of a novel ST9-spa type t4358-MRSA strain from pigs as well as pig handlers [91]. LA-MRSA ST9 is the primary sequence type in Asia, but this clone harbored different types of *SCCmec* in different countries, as follows: Type V in Malaysia, types IV and V in Taiwan, types II and IVb in China, and types IV, IVb, or V in Hong Kong [104]. More surveillance efforts are clearly needed to monitor the emergence of LA-MRSA in farm animals and animal handlers, as well as animal products, in view of their potential impact on human health. 

## 5. Conclusions

Our review of the prevalence of antimicrobial resistance in Malaysian *S. aureus* isolates obtained from the annual NSAR reports as well as various peer-reviewed published papers indicated an intriguing trend whereby the prevalence of resistance for the older line of antibiotics such as gentamicin, tetracycline and, to a lesser extent, erythromycin, has decreased over the past three decades. Chloramphenicol and co-trimoxazole also showed decreasing prevalence of resistance, whereas ciprofloxacin and rifampin retained almost the same level of prevalence despite fluctuations over the years. Only clindamycin showed a gradually increasing trend in the prevalence of resistance, although there was a nearly 20% decrease when comparing the prevalence in 2016 with 2017. So far, no Malaysian *S. aureus* isolates have been reported to be fully vancomycin resistant in peer-reviewed journals, although hVISA and VISA isolates have been reported. Some of the data from NSAR suggested an error in reporting (such as the chloramphenicol resistance data from 2008, which showed a massive ~80% increase in prevalence for that year only for the prevalence to decrease again to its pre-2008 levels the following years), but we were unable to assess the quality assurance or the validity of the data. Good quality surveillance data is essential in the global fight against the spread of antimicrobial resistance, hence the data and analysis provided by NSAR should be vastly improved. Molecular epidemiology data for *S. aureus* in Malaysia is still scarce and from the few published papers, the predominant HA-MRSA clone in Malaysia appeared to be ST239 with SCC*mec* type III whereas CA-MRSA isolates were mostly ST30 SCC*mec* type IV/V. As for LA-MRSA, the clones that have been reported so far were ST9 in pigs and ST692 in chickens. A comprehensive surveillance program which includes molecular epidemiological data should be established, which will enable us to have an in-depth understanding of the *S. aureus* clones that are circulating in the various healthcare institutions in Malaysia and to assess the extent of the spread of antimicrobial resistance among the *S. aureus* isolates. In our opinion, a more advanced and standardized tool such as WGS should be implemented in the government hospitals to obtain more comprehensive and better-quality surveillance data for Malaysia in order to further combat *S. aureus* infections in this country. This is feasible to be achieved one day if the current constraints, mainly due to the lack of financial and infrastructure support, can be overcome.

## Figures and Tables

**Table 1:** List of antimicrobials recommended by the joint commission of the European Centre for Disease Prevention and Control (ECDC) and the United States Centers for Disease Control and Prevention (CDC) for antimicrobial susceptibility testing of *S. aureus* [11]. The availability of standard breakpoints for the antimicrobials from CLSI or EUCAST and the antimicrobials reported by the Malaysian National Surveillance on Antimicrobial Resistance (NSAR) reports are indicated.

Antimicrobial Category	Antimicrobial Agent	CLSI Breakpoint	EUCAST Breakpoint	Reported by Malaysian NSAR
Aminoglycosides	Gentamicin	Yes	Yes	2005–2017
Ansamycins	Rifampin/rifampicin	Yes	Yes	2005–2017
Anti-MRSA cephalosporins	Ceftaroline	Yes	Yes	No
Anti-staphylococcal β-lactams (or cephamycins)	Oxacillin (or cefoxitin)	Yes	Yes	2003–2005
Fluoroquinolones	Ciprofloxacin	Yes	Yes	2007–2014
	Moxifloxacin	Yes	Yes	No
Folate pathway inhibitors	Trimethoprim-sulfamethoxazole	Yes	Yes	2005–2017
Fucidanes	Fusidic acid	No	Yes	2005–2017
Glycopeptides	Vancomycin	Yes	Yes	2003–2017
	Teicoplanin	Yes	Yes	2007–2008
	Telavancin	Yes	Yes	No
Glycylcyclines	Tigecycline	No	Yes	No
Lincosamides	Clindamycin	Yes	Yes	2005–2017
Lipopeptides	Daptomycin	Yes	Yes	No
Macrolides	Erythromycin	Yes	Yes	2005–2017
Oxazolidinones	Linezolid	Yes	Yes	2009–2017
Phenicols	Chloramphenicol	Yes	Yes	2005–2014
Phosphonic acids	Fosfomycin	No	Yes	No
Streptogramins	Quinupristin-dalfopristin	Yes	Yes	2011
Tetracyclines	Tetracycline	Yes	Yes	2009–2014
	Doxycycline	Yes	Yes	No
	Minocycline	Yes	Yes	No

CLSI, Clinical and Laboratories Standard Institute; EUCAST, European Committee on Antimicrobial Susceptibility Testing.

**Table 2:** Reported clinical *Staphylococcus aureus* studies in Malaysia.

Study (Period)	No. of *S. aureus* (*n*) or Percentage (%) of MRSA	Study Site(s) or No. of Study Site(s) (*n*)	Reference
Cheong et al., 1994 (1990–1991)	2586 35.5% (905) MRSA 539 MRSA included in study	HKL	[13]
Norazah et al., 2001 (1997–1999)	400 MRSA	Various, collected from ten hospitals throughout Malaysia	[14]
Al-Talib et al., 2010 (2002–2007)	8200 1979 MRSA	HUSM	[15]
Thong et al., 2009 (2003–2004 and 2007)	66 MRSA	N/A	[16]
Neela et al., 2008 (2006–2007)	32 MRSA	N/A	[17]
Lim et al., 2013 (2003 and 2008)	162 MRSA	UMMC	[18]
Al-Talib et al., 2015 (2008)	34 MRSA, 124 MSSA	HUSM	[19]
Noordin et al., 2016 (2009)	318 MRSA	UKMMC	[20]
Ho et al., 2017 (2011–2012)	175 MRSA	HRPB, KPJ and GP	[21]
Sit et al., 2018 (2013)	67 MRSA	UMMC	[22]
Che Hamzah et al., 2019 (2016–2017)	90 MRSA, 109 MSSA	HSNZ	[23]
NSAR (2003–2005)	N/A	N/A	[24]
NSAR (2006) **	12,370 [25] 31.5% MRSA [26]	10 GH [25]	[25,26]
NSAR (2007)	13,548 [25] 28% MRSA	12 GH [25,26]	[25,26]
NSAR (2008)	23,176 26% MRSA	13 GH	[25]
NSAR (2009)	20,053 21% MRSA	16 GH	[27]
NSAR (2010)	20,007 22.2% MRSA	16 GH	[28]
NSAR (2011)	31,140 19.2% MRSA	36 hospitals* (35 GH and 1 UH)	[29]
NSAR (2012)	32,611 17.3% MRSA	37 hospitals* (35 GH and 2 UH)	[30]
NSAR (2013)	34,492 17.7% MRSA	38 hospitals* (36 GH and 2 UH)	[31]
NSAR (2014)	37,341 17.2% MRSA	39 hospitals* (37 GH and 2 UH)	[32]
NSAR (2015)	37,416 19.3% MRSA	39 hospitals* (37 GH and 2 UH)	[33]
NSAR (2016)	37,207 18% MRSA	39 hospitals* (37 GH and 2 UH)	[4]
NSAR (2017)	39,447 19% MRSA	39 hospitals* (37 GH and 2 UH)	[5]

* Government hospitals (GH) and university hospitals (UH) distributed in all the 13 states in Malaysia; ** No antibiotic susceptibility profile available, the national MRSA rate and number of hospitals involved were obtained from the 2007 and 2008 NSAR reports [25,26]; MRSA, methicillin-resistant *S. aureus*; MSSA, methicillin-susceptible *S. aureus*; NSAR, National Surveillance of Antibiotic Resistance; HKL, Hospital Kuala Lumpur; Hospital HUSM, Hospital Universiti Sains Malaysia; UMMC, University of Malaya Medical Centre; UKMMC, Universiti Kebangsaan Malaysia Medical Centre; HRPB, Hospital Raja Permaisuri Bainun; KPJ, KPJ Ipoh Specialist Hospital; GP, Gribbles Pathology Ipoh; HSNZ, Hospital Sultanah Nur Zahirah; GH, government hospitals; UH, university hospitals; N/A, not available.

**Table 3:** Clonal characteristics of *Staphylococcus aureus* isolates in Malaysia.

**A. Community- and Hospital-Associated *Staphylococcus aureus* Isolates in Malaysia**					
**Year**	**MRSA/MSSA**	**SCC*mec* (%)**	**Sequence Type (ST)**	**Origin**	**Reference**
2003, 2004	MRSA	III (78.8) IV (18.2) Untypable (3.0)	N/A	N/A	[16]
2003, 2006, 2007	MRSA	IV IVE	ST6, ST30 ST22, ST1178, ST1179	HA, CA	[74]
2003, 2008	MRSA	III (90.0) IV (9.0) V (1.0)	(ST239, ST772, ST6, ST22, ST1178) *	HA, CA	[18]
2003, 2004, 2007, 2008	MRSA	NA	ST5, ST6, ST20, ST22, ST80, ST239	NA	[75]
2006–2007	MRSA	III (41.7) IIIA (52.8) V (5.5)	ST239 ST239 ST1, ST772	HA, CA	[68]
2006–2008	MRSA	III (96.8) IV (3.2)	NA ST30, ST22, ST45, ST80, ST101, ST188, ST1284, ST1285, ST1286, ST1287, ST1288	HA, CA	[69]
2007–2008	MRSA	III (20.0) IIIA (72.8) V (5.7) IVh (1.5)	(ST239, ST1, ST7, ST22, ST188, ST1283) *	NA	[67]
2008	MSSA	-	ST1, ST3, ST5, ST8, ST9, ST12, ST15, ST18, ST20, ST25, ST45, ST80, ST88, ST97, ST121, ST152, ST188, ST231, ST239, ST427, ST508, ST769, ST833, ST1050, ST1153	HA, CA	[76]
2008–2010	MRSA	III (91.4) IV (2.9) V (5.7)	(ST239, ST772) *	N/A	[77]
	MSSA	-	ST1, ST7, ST30, ST239, ST508, ST779, ST1179, ST1659		
2009	MRSA	II (0.4) III (94.5) IV (3.4) V (1.7)	ST239 ST239 ST30, ST1178 ST772	N/A	[78]
2009	MRSA	III (72.0) IV (2.5) V (1.3) II (0.3) Untypable (9.7) Novel (14.2)	N/A	NA	[20]
2010	MRSA	III (78.5) IV (21.5)	N/A	NA	[79]
2011–2012	MRSA	III (81.1) IV (12.6) II (0.6) V (5.7%)	N/A	NA	[21]
2011–2012	MRSA	II (0.9) III (66.5) IV (28.2) V (3.3) Untypable (0.9)	NA ST239 ST1, ST6, ST22, ST1137 ST5, ST772 ST239, ST508	HA, CA	[80]
2013	MRSA	III (55.2) I (1.5) IV (29.9) V (11.9) Untypable (1.5)	ST239 ST152 ST6, ST22, ST30, ST1179 ST1, ST45, ST772, ST951 ST5	HA, CA	[22]
2015–2017	MRSA	IVa (35.0) IVc (2.7) V (2.7) II (2.7) Untypable (57.0)	N/A	CA	[81]
**B. Livestock-Associated *Staphylococcus aureus* Isolates in Malaysia**					
**Year**	**Livestock**	**Prevalence (%)**	**SCC*mec* (%)/Sequence Type (ST)**	**Origin**	**Reference**
N/A	Raw chicken meat	*S. aureus* (24.0)	N/A	LA	[82]
N/A	Cat and dog	MRSA (11.7)	N/A	LA	[83]
N/A	Cat and dog	MRSA (1.9)	N/A	LA	[84]
N/A	Chicken	MRSA (18.0)	N/A	LA	[85]
N/A	Piglet	MRSA (2.4)	N/A	LA	[86]
2007–2008	Cat and dog	MRSA (8.0)	N/A	LA	[87]
N/A	Horse	*S. aureus* (44.0)	N/A	LA	[88]
2010	Chicken	*S. aureus* (1.0)	ST692	LA	[89]
2014	Cat and dog	MRSA in cat (7.7) MRSA in dog (11.7)	N/A	LA	[90]
N/A	Pig	MRSA (1.4)	V (100.0)/ST9	LA	[91]
N/A	Pig	*S. aureus* (24.6) MRSA (0.8)	N/A	LA	[92]

* Sequence type not correlated with SCC*mec* type; ST, sequence type; MRSA, methicillin-resistant *S. aureus*; MSSA, methicillin-susceptible *S. aureus*; CA, community-associated; HA, hospital-associated; LA, livestock-associated; N/A, not available.

## Abbreviations

ACME	Arginine Catabolic Mobile Element
CA	community-associated
CC	clonal complex
CDC	Centers for Disease Prevention and Control
CLSI	Clinical and Laboratories Standard Institute
cMLS_B_	constitutive macrolide-lincosamide-streptogramin B
ECDC	European Centre for Disease Prevention and Control
EUCAST	European Committee on Antimicrobial Susceptibility Testing
GP	Gribbles Pathology Ipoh
HA	hospital-associated
HKL	Hospital Kuala Lumpur
HRPB	Hospital Raja Permaisuri Bainun
HSNZ	Hospital Sultanah Nur Zahirah
hVISA	heterogeneous vancomycin-intermediate *S. aureus*
HUSM	Hospital Universiti Sains Malaysia
iMLS_B_	inducible macrolide-lincosamide-streptogramin B
IMR	Institute for Medical Research
KPJ	KPJ Ipoh Specialist Hospital
LA	livestock-associated
MIC	minimum inhibition concentration
MLS_B_	macrolide-lincosamide-streptogramin B
MRSA	methicillin-resistance *S. aureus*
MS	macrolides streptogramin
MSSA	methicillin-susceptible *S. aureus*
MLST	multilocus sequence typing
MyOHAR	Malaysian One Health Antimicrobial Resistance
NSAR	National Surveillance on Antimicrobial Resistance
PBP 2a	penicillin binding protein 2a
PVL	panton-valentine leucocidin
SCC*mec*	staphylococcal cassette chromosome *mec*
ST	sequence type
UKMMC	Universiti Kebangsaan Malaysia Medical Centre
UMMC	University of Malaya Medical Centre
VISA	vancomycin-intermediate *S. aureus*
VRSA	vancomycin-resistant *S. aureus*
WGS	whole genome sequencing
WHO	World Health Organization
